# Walnut Protein Hydrolysates Play a Protective Role on Neurotoxicity Induced by d-Galactose and Aluminum Chloride in Mice

**DOI:** 10.3390/molecules23092308

**Published:** 2018-09-10

**Authors:** Li Feng, Xiaojing Wang, Fei Peng, Jianqiao Liao, Yifan Nai, Hongjie Lei, Mei Li, Huaide Xu

**Affiliations:** College of Food Science and Engineering, Northwest A&F University, Yangling 712100, China; fengli0304@163.com (L.F.); wangxj0930@163.com (X.W.); 15029292654@163.com (F.P.); ljq1150163714@163.com (J.L.); 2014014831@nwsuaf.edu.cn (Y.N.); leihongjie@nwafu.edu.cn (H.L.)

**Keywords:** walnut protein hydrolysates, gastrointestinal digestion, d-galactose, aluminum chloride, neurotoxicity, antioxidant activity

## Abstract

In recent years, with an increase in the aging population, neurodegenerative diseases have attracted more and more attention. This study aimed to investigate the potential neuroprotective effect of defatted walnut meal protein hydrolysates (DWMPH) on neurotoxicity induced by d-galactose (d-gal) and aluminum chloride (AlCl_3_) in mice. The animal models were established by combining treatments with d-gal (200 mg/kg/day, subcutaneously) and AlCl_3_ (100 mg/kg in drinking water) for 90 days. During the 90 days, 1 g/kg of DWMPH was administrated orally every day. The results indicated that DWMPH treatment alleviated oxidative stress, reversed cholinergic dysfunction, and suppressed the release of proinflammatory cytokines in the brains of d-gal + AlCl_3_-treated mice, and thus improving the learning and memory functions of these mice, which was closely correlated with the strong antioxidant activity of DWMPH. This finding suggests that DWMPH might be a promising dietary supplement in improving neuronal dysfunctions of the brain.

## 1. Introduction

It has been recognized that free radicals and oxidative stress play a vital role in neuronal degeneration, probably because the brain is more vulnerable to damage by oxidative stress than other organs, for its high rate of oxygen consumption, abundance of unsaturated lipids, and relatively lower availability of antioxidant enzymes [1,2]. Additionally, free radicals could trigger inflammatory responses in central nervous systems (CNS), including an elevation of tumor necrosis factor α (TNF-α) and interleukin 1β (IL-1β) levels [3]. The release of neuroinflammation has been proven to be associated with the pathogenesis of several neurodegeneration, such as Alzheimer disease (AD) and Parkinson disease [4,5]. Therefore, possible therapeutic and preventive strategies have always been attracted to the inhibition of oxidative stress and inflammation, which is based on their pivotal roles in the pathogenesis of neuronal degeneration.

Chronic exposure to d-galactose (d-gal), which is widely used to establish a model of accelerated aging, could induce oxidative stress, influence neurogenesis, and then, result in neurodegeneration, similar to the natural aging in mice [6]. Additionally, aluminum, a neurotoxic agent, could induce cholinergic dysfunction in the CNS and the generation of free radicals and neurotoxicity in the brain [7,8]. Recently, mice administered with 60 mg/kg d-gal and 10 mg/kg aluminum chloride (AlCl_3_) intraperitoneally for 40 days eventually led to an abnormal cholinergic system, oxidative stress, neuronal apoptosis, and neuroinflammation, accompanied by cognitive impairment [9]. Consequently, chronic d-gal and AlCl_3_ combination stimulation has become a useful model to study ageing and ageing-related neurodegenerative diseases and drug screening.

Walnut is commonly known as ‘brain food’ for its abundant lipids, proteins, carbohydrates, vitamins, and minerals [10]. Although the dietary supplementation of walnuts has been proven to improve memory deficits and learning skills in transgenic mice models, many researchers still pay more attention to walnut oils, which are considered to be rich in polyunsaturated fatty acids with a strong neuroprotective effect [11,12]. In recent years, it has been found that the peptides from different kinds of protein hydrolysates possessed potential bioactivities, such as hazelnut peptides against oxidative stress, and oyster peptides, which improved learning and memory of mice [13,14]. Moreover, gastrointestinal digestion has been suggested as an effective pathway to produce bioactive peptides, which have been used to prepare bioactive peptides from chicken breast and rapeseed proteins [15,16,17]. To date, walnut protein hydrolysates prepared using a mixture of pancreatin and viscozyme L have been proven to possess neuroprotective effects [18]. However, few studies were focused on the neuroprotective effect of peptides from walnut protein or defatted walnut meal protein by simulated gastrointestinal digestion. In the present study, we investigated the potential neuroprotective effect of defatted walnut meal protein hydrolysates (DWMPH) on neurotoxicity induced by d-gal and AlCl_3_ combination in Kunming mice, by examining the cognitive capacity of experimental mice and assessing the oxidative stress, cholinergic function, and neuroinflammatory of mice brains. Furthermore, we examined the structure and antioxidant activity of DWMPH. Finally, the possible mechanism for the neuroprotective activity of DWMPH was elucidated.

## 2. Results and Discussion

### 2.1. Body Weight and Food Intake

To investigate the weight or appetite of each group of mice, the body weight and food intake were recorded during the experiment. As shown in Table 1, the initial body weight, final body weight, and food intake did not show significant differences among all of the groups, indicating that d-gal and AlCl_3_ or DWMPH did not affect the body weight and appetite of mice.

### 2.2. Effect of DWMPH on Mice Behavioral Test

**Figure 1:**
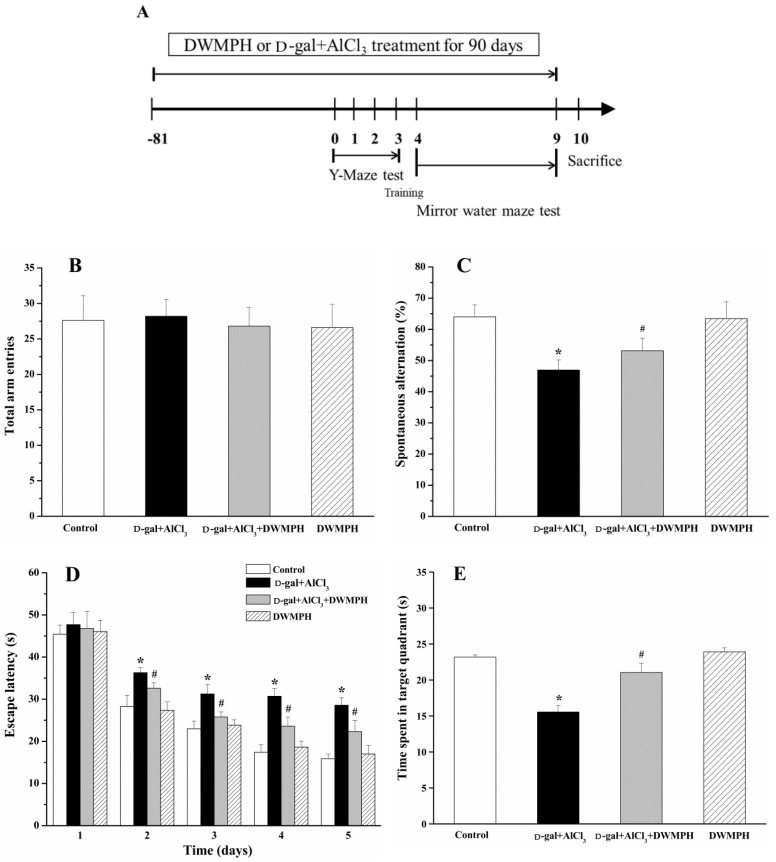

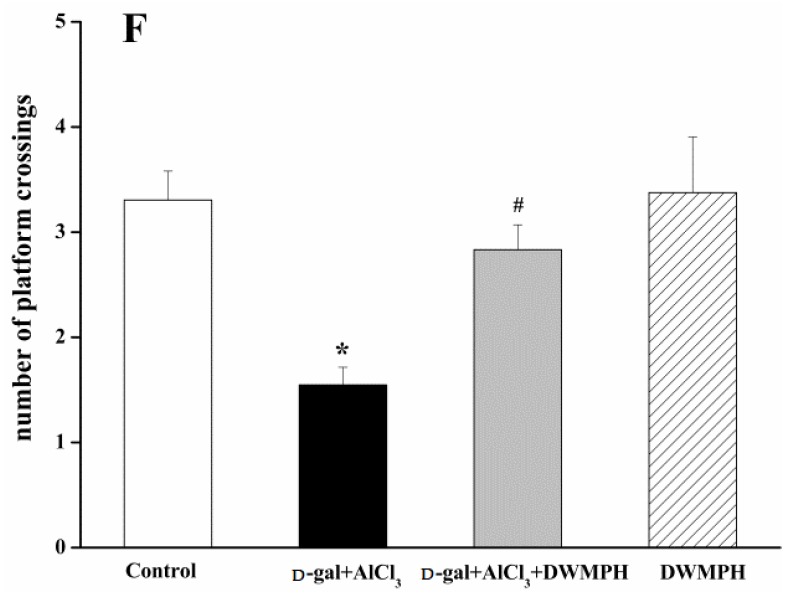
Timeline illustrates establishment of mice model, defatted walnut meal protein hydrolysates (DWMPH) treatment and assessments of cognitive functions of mice. (**A**) Experimental schedule for investigating the effect of DWMPH on cognitive impairment induced by d-galactose (d-gal) and aluminum chloride (AlCl_3_). Effect of DWMPH on total arm entries (**B**) and spontaneous alternation (**C**) in Y-maze task were recorded. Escape latency (**D**), time spent in target quadrant (**E**), and number of platform crossings (**F**) in the Morris water maze test were also recorded. The data are expressed as means ± standard deviation (SD). * *p* < 0.05 vs. control group; ^#^ *p* < 0.05 vs. d-gal + AlCl_3_ group.

Y-maze task and Morris water maze test (MWM) were performed as scheduled (Figure 1A). As shown in Figure 1B, there were no significant differences among the four groups in the total arm entries, showing that d-gal, AlCl_3_, or DWMPH did not affect the motor activities of mice. Spontaneous alteration is usually used to analyze the spatial working memory. The mice with a higher percentage of spontaneous alteration were considered to have a better working memory. The mice treated with d-gal and AlCl_3_ showed a lower percentage of spontaneous alteration than that of the control group, while the percentage of spontaneous alteration was significantly increased by DWMPH treatment in the d-gal+AlCl_3_-treated mice, compared to the d-gal+AlCl_3_-treated-alone mice, indicating that DWMPH could attenuate the loss of working memory (Figure 1C).

Following the Y-maze test, we performed a MWM test to investigate the effect of DWMPH on spatial memory of mice. As illustrated in Figure 1D, the mice treated with d-gal and AlCl_3_ took a longer time to find the platform than that of the control group, while the DWMPH treatment to d-gal + AlCl_3_-treated mice showed less escape latency than that of the d-gal + AlCl_3_ group. After the removal of the hidden platform, a probe test was performed. Mice stimulated by d-gal and AlCl_3_ spent less time in the target quadrant and a lower number of platform crossings than that of the control group, whereas the DWMPH treatment markedly increased the time spent in target quadrant and the number of platform crossings in the d-gal + AlCl_3_-treated mice compared to the d-gal + AlCl_3_-treated-alone mice (Figure 1E,F). These results indicated that DWMPH reduced the spatial memory impairment induced by d-gal and AlCl_3_.

The results of the Y-maze and MWM test showed that the learning and memory abilities of the d-gal + AlCl_3_-treated mice were significantly decreased compared to the control group, which was consistent with the previous report that mice that were treated long-term with both d-gal and AlCl_3_ eventually damaged their learning and memory functions [9]. Notably, DWMPH attenuated the impairment of learning and memory abilities.

### 2.3. Effect of DWMPH on Neuronal Morphology

**Figure 2:**
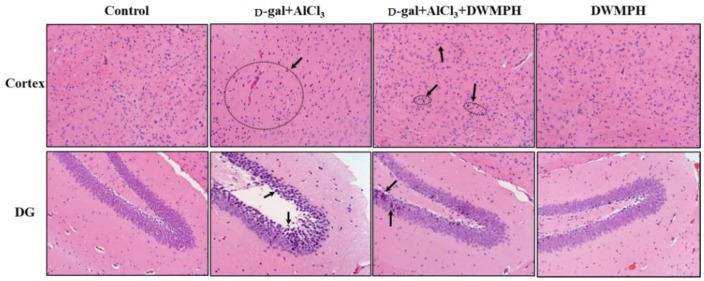
Effect of DWMPH on the changes of neuronal morphology induced by d-gal and AlCl_3_. Brain tissues (cortex and hippocampus) stained with hematoxylin-eosin (HE). The arrows indicate the apoptotic neurons.

Sustaining oxidative stress and metal ions are considered to be associated with the development of neurodegenerative diseases, and could lead to tissue damage [2,19,20]. Hematoxylin-eosin (HE) staining was performed to observe the neuroprotection of DWMPH on the cortex and hippocampus damaged by d-gal and AlCl_3_. As shown in Figure 2, the brain tissues of the d-gal + AlCl_3_-treated mice exhibited many pyknotic nuclei. Compared with the control group, the neurons were markedly shrunken and the density of neurons was decreased in the cortex and in the hippocampal dentate gyrus (DG) regions, which was in agreement with the neuronal abnormalities that occurred in other nerve impairment models [21,22]. Remarkably, the DWMPH treatment to d-gal + AlCl_3_-treated mice reversed this damage, and the pyramidal cells in the hippocampal DG regions were shown to arrange more orderly and tightly with less neuronal loss than the d-gal + AlCl_3_-treated group, which was consistent with the results of behavior tests above.

### 2.4. DWMPH Attenuate Oxidative Damage and Reverse Cholinergic Dysfunction

**Figure 3:**
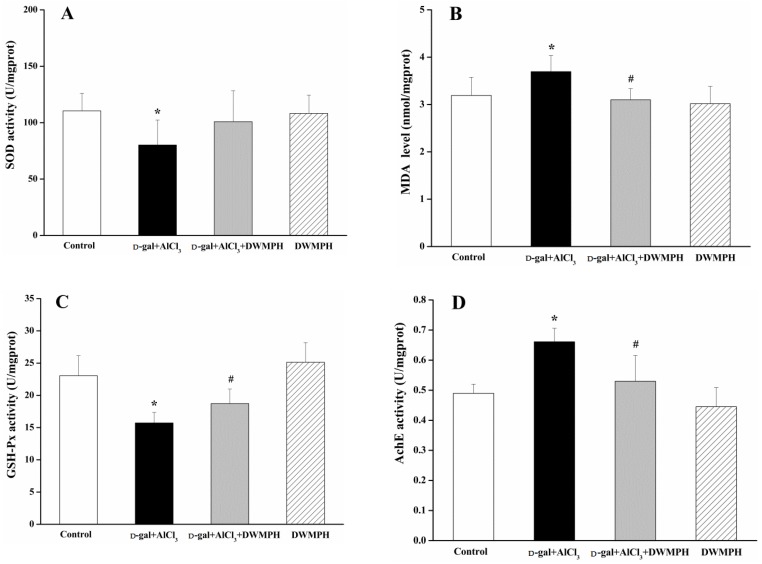

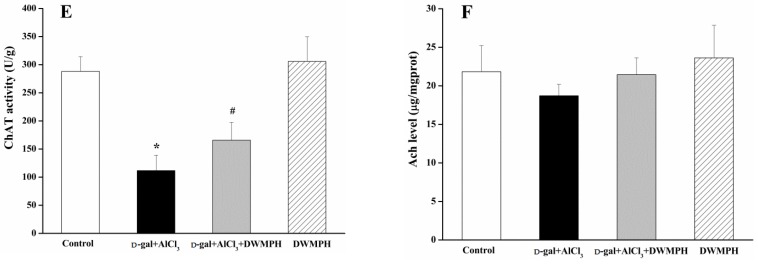
Alleviation of DWMPH on oxidative damage and cholinergic dysfunction in mice brain. The activities of superoxide dismutase (SOD) (**A**) and glutathione peroxidase (GSH-Px) (**C**), as well as the content of malondialdehyde (MDA) (**B**) in brains, were measured. The acetylcholine esterase (AChE) activity (**D**), choline acetyltransferase (ChAT) activity (**E**), and levels of acetylcholine (ACh) (**F**) related to cholinergic dysfunction in the mouse brain were also determined. The data are expressed as means ± SD. * *p* < 0.05 vs. control group, ^#^ *p* < 0.05 vs. the d-gal + AlCl_3_ group.

As one of the important factors contributing to pathogenesis in neurological diseases, oxidative damage is generally determined through the contents of superoxide dismutase (SOD), glutathione peroxidase (GSH-Px), and malondialdehyde (MDA). As shown in Figure 3A–C, the contents of SOD and GSH-Px were decreased in the brain tissues of the d-gal + AlCl_3_-treated group compared with the control group. The MDA level was significantly higher in the d-gal + AlCl_3_-treated group than that in the control group. These results were in accordance with previous findings, that the activities of SOD and GSH-Px were decreased while the MDA level was raised in the brains of mice with cognitive deficits [2]. In addition, the levels of SOD, GSH-Px, and MDA are related to oxidative stress, among which SOD and GSH-Px are endogenous antioxidant enzymes and MDA is a key indicator of lipid peroxidation. It has been reported that the balance of the cholinergic system is also interrelated with the cognitive function in neurodegenerative diseases [23,24]. As illustrated in Figure 3D–F, compared to the control group, the contents of choline acetyltransferase (ChAT) and acetylcholine (ACh) were decreased and the acetylcholine esterase (AChE) activity was markedly increased in the brain tissues of the d-gal + AlCl_3_-treated group. Ach, an essential neurotransmitter, is synthesized from choline and acetyl-CoA via the stimulation of ChAT, and hydrolyzed by AChE, to generate acetate and choline in the synaptic cleft. The increase of the Ach level was associated with cognitive improvement [25]. Contrastingly, the contents of SOD, GSH-Px, ChAT, and Ach were increased and significant differences were observed in the GSH-Px and ChAT levels, whereas the levels of MDA and AChE were markedly decreased in the brain tissues of the DWMPH + d-gal + AlCl_3_-treated group compared to those of the d-gal + AlCl_3_-treated group. The above results showed that the brains of mice suffered from oxidative damage and might lead to cholinergic dysfunction after stimulated by d-gal and AlCl_3_, which could be reserved by DWMPH treatment.

### 2.5. DWMPH Reduce the Expressions of Inflammatory Factors TNF-α and IL-1β

**Figure 4:**
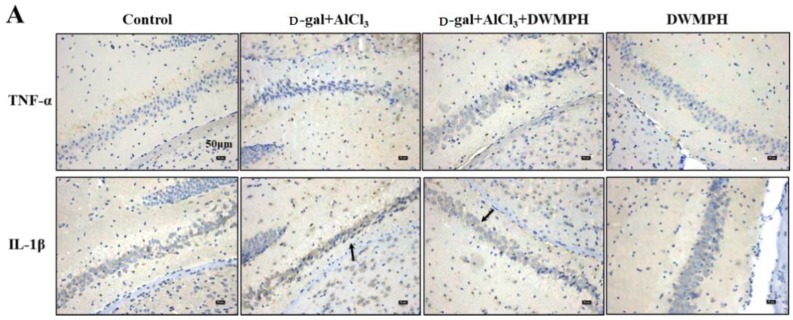

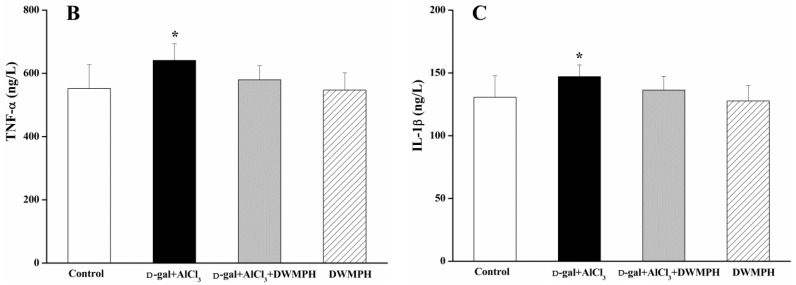
DWMPH reduces the expressions of inflammatory factors tumor necrosis factor α (TNF-α) and interleukin 1β (IL-1β). (**A**) Hippocampus stained by the immunohistochemical approach. Representative photomicrographs of the TNF-α and IL-1β expressions are shown. The levels of TNF-α (**B**) and IL-1β (**C**) were also measured by enzyme-linked immunoassay (ELISA). Data are expressed as means ± SD. * *p* <0.05 vs. control group.

Many factors potentially contribute to the risk of age-associated neuronal dysfunction, including oxidative damage and inflammatory induced by d-gal and AlCl_3_. In the present study, the inflammatory response was examined through the expressions of TNF-α and IL-1β. Immunohistochemical (IHC) staining was carried out to investigate the expressions of TNF-α and IL-1β and their cellular localization in mice brains. As shown in Figure 4A, a higher expression of TNF-α and IL-1β in the hippocampus were observed in the d-gal + AlCl_3_-treated group compared to the control group. After the DWMPH treatment, the expressions of TNF-α and IL-1β decreased in the DWMPH + d-gal + AlCl_3_-treated mice compared with the d-gal + AlCl_3_-treated mice. In addition, the enzyme-linked immunoassay (ELISA) results also showed that DWMPH down-regulated the expressions of TNF-α and IL-1β in mice brains induced by d-gal and AlCl_3_ (Figure 4B,C), indicating that DWMPH could reduce the inflammation stimulated by d-gal and AlCl_3_ in mice brains. Past works have described that an increased production of reactive oxygen species (ROS) in the brain leads to inflammatory mediators. Moreover, as the number of activated microglia cells and astrocytes increased, a variety of proinflammatory cytokines, including TNF-α and IL-1β were released, which are involved in synaptic plasticity and neurogenesis resulting in cognitive impairment [26,27,28]. Therefore, the possible mechanism was that DWMPH decreased ROS and then suppressed the expression of TNF-α and IL-1β in the brains of d-gal + AlCl_3_-treated mice, speculating that DWMPH could possess strong antioxidant activity. 

### 2.6. Structure and Antioxidant Activity of DWMPH

The structural changes of DWMPH prepared by simulated gastrointestinal digestion from DWMP were evaluated by amino acid compositions, secondary structures, and molecular weight (MW). In addition, 2,2-azino-bis (3-ethylbenzothiazoline-6-sulfonic acid) (ABTS) radical scavenging activity and oxygen radical absorbance capacity (ORAC) assays were used to verify the antioxidant activity of DWMPH in vitro.

Amino Acid Compositions. As shown in Table 2, the contents of tryptophan (Trp), Asp, and Lys in DWMPH were higher than that of the defatted walnut meal proteins (DWMP). Amino acid compositions of the peptides play a vital role in the antioxidant activity. It has been reported that peptides containing Trp residue exhibit good antioxidant activities for their phenolic hydroxyl group, which could scavenge the free radicals via donating protons to electron deficient radials [29]. In addition, the increase of Asp and Lys may contribute to the antioxidant capacity of DWMPH for the charged residues of them [30]. This finding implied that DWMPH might possess a potential antioxidant activity.

**Figure 5:**
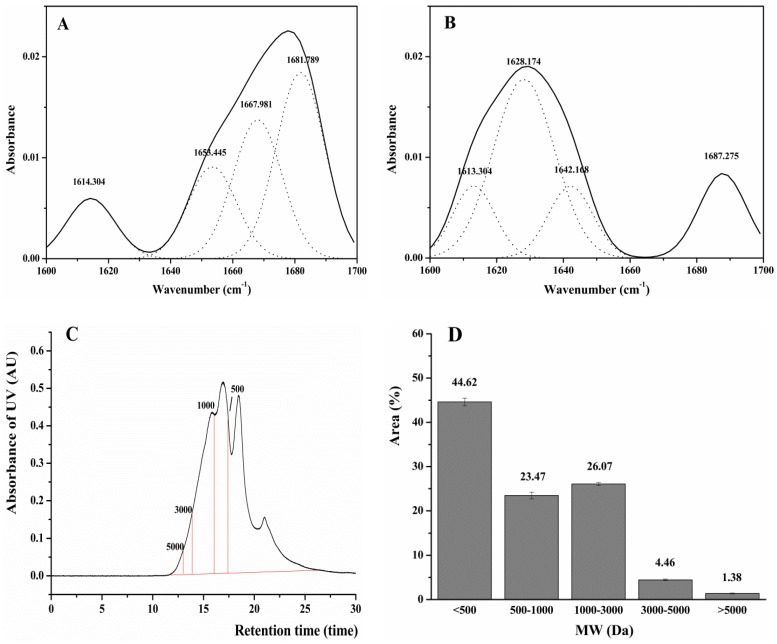

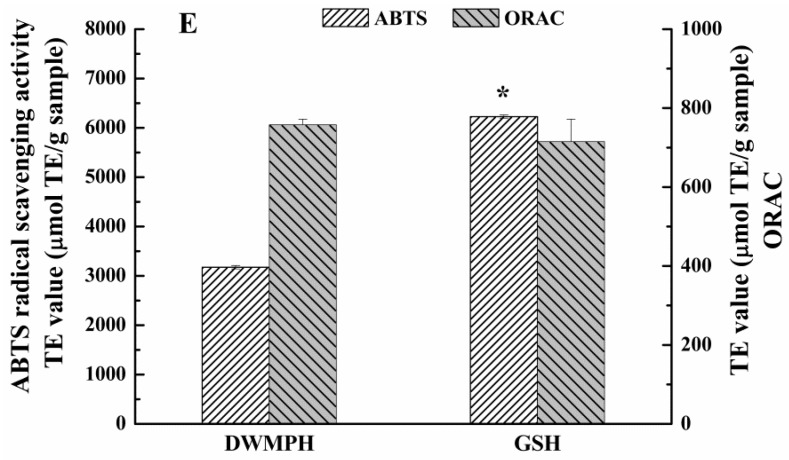
The chemical characteristics of DWMPH. The FTIR spectra of DWMPH (**B**) compared with DWMP (**A**). (**C**) The chromatogram of DWMPH by gel chromatography. (**D**) The molecular weight (MW) distribution of DWMPH. (**E**) Antioxidant activities of DWMPH evaluated by 2,2-azino-bis (3-ethylbenzothiazoline-6-sulfonic acid) (ABTS) radical scavenging activity and oxygen radical absorbance capacity (ORAC) compared to that of GSH. Data are expressed as means ± SD. * *p* < 0.05 vs. DWMPH.

Secondary Structures and MW Distribution of DWMPH. In this work, FTIR was used to investigate the change in the secondary structure of DWMPH compared with DWMP. The characteristic bands of the proteins and peptides in the FTIR spectra are mainly included in the amide I region (1600–1700 cm^−1^). As shown in Figure 5A, the secondary structure of DWMP was composed of α-helixes (1653 cm^−1^), β-turns (1667 cm^−1^), and β-sheets (1614 cm^−1^ and 1681 cm^−1^). The percentage of these secondary structures are in the order of β-sheets > β-turns > α-helixes, suggesting that the β-sheets were the dominant secondary structure in DWMP. However, it could be seen that the secondary structure of the α-helixes disappeared, and the content of β-turns (1687 cm^−1^) was largely decreased, while random coils (1642 cm^−1^) emerged in the DWMPH (Figure 5B). This result was in agreement with the previous research, that the β-turn structure largely disappeared and the content of the irregular structure was increased in the peanut protein hydrolysates [31]. Furthermore, a loss of α-helixes is considered to be strongly connected with the increased antioxidant activity of peptides [32]. Based on the changes of the secondary structure, it could be speculated that the polypeptide chains in DWMP were ongoing significant denaturation, and the α-helixes of the DWMP transformed into random coils after enzymatic hydrolysis. With the exposure of the active sites, DWMPH may exhibit stronger antioxidant capacity.

Gel chromatography was used to study the MW distribution of DWMPH, and the results are shown in Figure 5C,D. DWMPH were rich in the fractions, with molecular weight lower than 3000 Da. The proportions of 1000–3000 Da, 500–1000 Da, and <500 Da were 25.66%, 22.60%, and 45.62%, respectively. That is to say, the DWMPH were mainly composed of fractions with MW <3000 Da, indicating that DWMP was greatly degraded by simulated gastrointestinal digestion, in accordance with the result of secondary structures. Moreover, it has been demonstrated that the antioxidant bioactivity of the protein hydrolysates were highly correlated with MW, and the peptides with a smaller size possessed a higher antioxidant activity and other functional properties [33,34]. Hence, DWMPH might possess antioxidant activity.

Antioxidant Activities of DWMPH. To verify the DWMPH’s antioxidant activity, the ABTS radical scavenging activity and ORAC assays were used in this study. As shown in Figure 5E, the strong capacity of DWMPH in the scavenging ABTS radicals was 3175.70 µmol TE/g, and the ORAC value similar with GSH was 757.67 µmol TE/g. This result showed that DWMPH possesses excellent antioxidant activity, which was in accordance with the results of the amino acid compositions and the MW distribution.

Of course, the antioxidant activity of the peptides not only depends on the amino acid compositions and MW distribution, but also on other variables such as the enzymatic type. It has been reported that pepsin hydrolyzes protein mainly at a carboxyl group provided by aromatic amino acids [35]. Therefore, aromatic amino acids might present at the N-terminus of peptides from pepsin hydrolysates and act as antioxidant contributors. In addition, pepsin firstly broke DWMP into small peptides, and then pancreatin digested these peptides into a smaller size during in vitro gastrointestinal digestion, which can be easily absorbed and interacted with radicals more effectively [16,36,37,38].

## 3. Materials and Methods

### 3.1. Materials

Defatted walnut meal was obtained from Shaanxi Sea Ecological Agriculture Co., Ltd. (Shangluo, China). Pancreatin was purchased from Novozymes (Beijing, China). Pepsin was the product of Sigma-Aldrich (St. Louis, MO, USA). d-gal was obtained from Solarbio Science and Technology Co., Ltd. (Beijing, China). All of the other chemicals and reagents used were of analytical grade.

### 3.2. Preparation of Defatted Walnut Meal Protein Hydrolysates (DWMPH)

The DWMPH was prepared according to the method of a previous study [39]. After centrifugation, the supernatants of the reaction system were concentrated below 50 °C and then dialyzed with dialysis membranes of 100 Da to remove small molecules at 4 °C. The samples were lyophilized and stored at −20 °C until use. The yields of DWMP and DWMPH from defatted walnut meal were approximately 23% and 9.8%, respectively.

### 3.3. Animals and Treatments

Male wild type Kunming mice (25–30 g, 8 weeks old) were purchased from Xi’an Jiaotong University (Xi’an, China). The mice were acclimatized for 1 week in the animal house, under standard conditions (12/12 h light–dark cycle, humidity at 50 ± 15%, temperature 22 ± 2 °C) and allowed free access to food and water ad libitum.

The mice were randomly divided into the following four groups (*n* = 10 per group): control, d-gal + AlCl_3_, d-gal + AlCl_3_ + DWMPH, and DWMPH groups. The mice in the d-gal + AlCl_3_ + DWMPH and DWMPH group were administered intragastrically with DWMPH (1 g/kg per day, dissolved in distill water) for 90 days. The control and d-gal + AlCl_3_ group mice were administered intragastrically with the same volume of distilled water. Six hours later, the mice in the d-gal + AlCl_3_ and d-gal + AlCl_3_ + DWMPH group were subcutaneously injected with 200 mg/kg/day d-gal in normal saline for 90 days. For the control and DWMPH group, the mice were subcutaneously injected with the same volume of sterile saline alone. During the 90 days, the mice in the d-gal + AlCl_3_ and d-gal + AlCl_3_ + DWMPH group received 100 mg/kg AlCl_3_ in their drinking water. The body weight was recorded every three days and the food intake was measured once a week. Y-maze task and Morris water maze were used to evaluate the learning and memory capability in mice. After the behavioral tests, the mice were humanely killed, and the brain tissues were collected quickly, frozen in liquid nitrogen, and stored at −80 °C.

This study was carried out strictly following to the eighth edition of ‘Guide for the Care and Use of Laboratory Animals’ of the National Institutes of Health, and all of the experimental procedures were approved by the Animal Ethics Committee of Xi’an Jiaotong University (SCXK [shan] 2012-003).

### 3.4. Y-Maze Task

The Y-maze was performed to determine the learning and memory capabilities. The Y-maze sets were constructed with three black arms converged to an equal angle, and each arm was 35 cm long, 15 cm high, and 6 cm wide (Shanghai Xinruan Information Technology, Shanghai, China). Each mouse was placed at the center of the apparatus and allowed to explore the maze freely for 8 min. Spontaneous alteration was defined as the successive entry into three different arms. The total number of arm entries and alternation were recorded respectively. The percentage alternation (%) was calculated as (the number of alternation/[total number of arm entries − 2]) × 100%.

### 3.5. Morris Water Maze Test

The Morris water maze test (MWM) was performed to examine the spatial memory of the mice. The apparatus consisted of a circular water tank (150 cm in diameter and 35 cm in height) filled with nontoxic water (23 ± 1 °C), which was rendered opaque by adding brilliant black. The tank was divided into four quadrants and a black escape platform was placed at the midpoint of one quadrant, which was hidden 1 cm below the surface of water. The test consisted of five days of training trials and a one-day probe trial. Each mouse received training per day for five consecutive days, using a single hidden platform in one quadrant, with three quadrants of rotational starting. After the mouse found and climbed onto the platform within 60 s, the training was stopped and the escape latency was recorded. If the mouse failed, it was guided to the platform by a stick and allowed to remain on the platform for 30 s. The escape latency of 60 s was recorded. About 24 h after the last training session, a probe trial, during which the escape platform was removed from the maze, was conducted to assess the spatial memory. The time spent in the target quadrant and the times of crossing the former platform location were recorded.

### 3.6. Measurement of Cytokine, Level of Ach, and MDA, and Activities of GSH-Px, SOD, AChE, and ChAT

Brain tissues taken from the deep-freezer were weighed and homogenized in a nine-fold volume of saline. The homogenates were then centrifuged at 4000 rpm for 15 min at 4 °C. The supernatants were used for biochemical analysis. The contents of acetylcholine (ACh) and malondialdehyde (MDA), as well as the activities of glutathione peroxidase (GSH-Px), superoxide dismutase (SOD), acetylcholine esterase (AChE), and choline acetyltransferase (ChAT) in the mouse brains were measured using commercial kits from Nanjing Jiancheng Bioengineering Institute (Nanjing, China), according to the manufacturer’s protocol. In addition, the level of TNF-α and IL-1β in the mouse brains were detected using commercial enzyme-linked immunoassay (ELISA) kits purchased from Xinle Biology Technology (Mouse TNF-α kit, Mouse IL-1β kit; Shanghai, China).

### 3.7. Hematoxylin-Eosin and Immunohistochemical Staining

After being deeply anesthetized with chloral hydrate, the mice were perfused transcardially with saline, and the isolated brain tissues were postfixed in 4% paraformaldehyde. The brain sections containing the hippocampus from the fixed tissues were dehydrated and then embedded in paraffin. The brain sections were cut into 5 μm-thin sections, dewaxed, and rehydrated using xylene and declining grades of ethanol. Subsequently, the brain slices were stained with hematoxylin-eosin (HE).

The dewaxed and rehydrated slices were also dyed using an immunohistochemical approach, to visualize the expressions of TNF-α and IL-1β in the mice brains. Briefly, the slices were boiled to perform antigen retrieval after permeabilized by Tris-EDTA buffer (pH 9.0). Then, endogenous peroxidases were quenched from the tissues by 3% H_2_O_2_. After being blocked with a normal goat serum blocking solution for 20 min, the slices were incubated with primary antibodies of rabbit anti-TNF-α and anti-IL-1β (Cell Signaling Technology, Danvers, MA, USA) overnight at 4 °C. After washing three times with PBS, the slices were incubated with goat anti-rabbit secondary antibody for 20 min at 37 °C, and subsequently reacted with horseradish peroxidase-streptavidin (Streptavidin Peroxidase Link Detection Kits; Beijing Zhongshan Golden Bridge Biotechnology Co., Ltd., Beijing, China) for 20 min at room temperature. The slices were then visualized by the chromogen 3,3’-diaminobenzidine tetrahydrochloride (DAB) kit (Beijing Zhongshan Golden Bridge Biotechnology Co., Ltd.).

### 3.8. Amino Acid Compositions, Secondary Structures And Molecular Weight Distribution of DWMPH

The amino acid compositions of DWMP and DWMPH were determined according to the method of Su et al. (2011), with some modification [40]. DWMP and DWMPH were hydrolyzed with 6 M HCl at 110 °C for 24 h, followed by derivatization with o-Phthalaldehyde, respectively. To determine the level of tryptophan (Trp), the alkaline hydrolysis of DWMP and DWMPH was also conducted with 4 M NaOH at 105 °C for 24 h. Amino acid analyses were performed using reverse phase high-performance liquid chromatography (RP-HPLC) equipped with a Hypersil ODS column (4.0 mm × 250 mm, 5 μm, Agilent Technologies, Santa Clara, CA, USA). Amino acid standard solutions were used as external standards and the contents of the individual amino acids were expressed as g/100 g protein.

The change in the secondary structures of DWMPH compared with DWMP was analyzed by Fourier transform infrared spectrophotometer (FTIR, Bruker, Germany), according to the method reported by Duan [41]. Briefly, the dried samples were mixed with KBr in ratio of 1:200, and milled to fine powders in a mortar with a pestle. After the powders were pressed into thin sheets under a pressure of 4 t, the spectra of the samples were recorded against an air background in range of 4000–400 cm^−1^, and analyzed with PeakFit v4.12 (SeaSolve Software Inc., Framingham, MA, USA).

The MW distribution of DWMPH was determinated by gel chromatography, according to the previously described method, with some modifications [41]. A Waters 1525 HPLC system with a silica-based column (TSK G2000SWXL, 300 × 7.8 mm, Tokyo, Japan) was used for the analysis. All of the fractions were successively eluted by mobile phase (Acetonitrile:distilled water:trifluoroacetic acid = 40:60:0.1, *v*/*v*/*v*) at a flow rate of 0.5 mL/min. The absorbance was monitored at 214 nm. Four protein standards, cytochrome c (12,384 Da), bacitracin (1422.69 Da), Gly-Gly-Tyr-Arg (451.48 Da), and Gly-Gly-Gly (189.17 Da), were used to make a reference curve. Then, the MW was calculated by the elution volume.

### 3.9. Assays of Antioxidant Activity

The antioxidant capacity of DWMPH was evaluated by the ABTS radical scavenging activity, and ORAC assays were conducted following the methods described in a previous study [39].

### 3.10. Statistical Analysis

All of the results were expressed as means ± standard deviation (SD). The data were analyzed by one-way ANOVA using SPSS 18.0, and a least significant difference (LSD) range test was conducted to identify the significance of the differences (*p* < 0.05).

## 4. Conclusions

**Figure 6:**
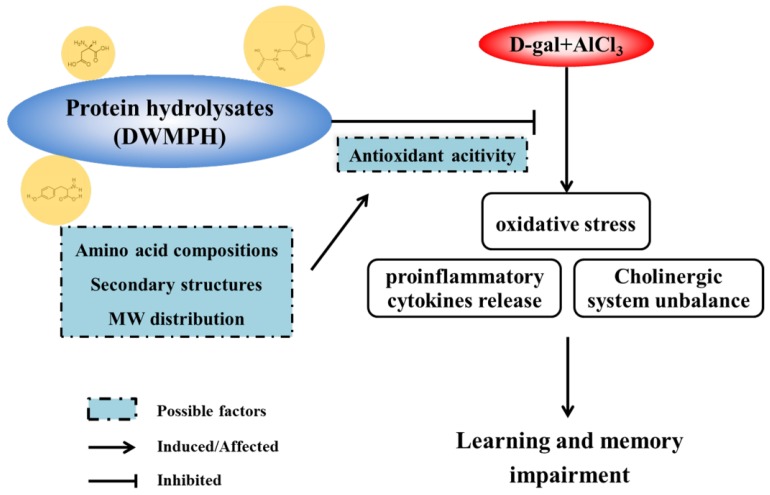
The mechanism of DWMPH on alleviation of d-gal and AlCl_3_ induced neurotoxicity in mice.

In conclusion, this study investigated the protective effect of DWMPH on neurotoxicity induced by d-gal and AlCl_3_ in mice. The results indicated that the DWMPH treatment attenuated d-gal + AlCl_3_-induced neuronal dysfunction through alleviating oxidative stress, reversing cholinergic dysfunction, and suppressing the release of proinflammatory cytokines in the brains of d-gal + AlCl_3_-treated mice, which were evidenced by increased the activities of SOD, GSH-Px, and ChAT, and the decreased the levels of MDA, Ach, AChE, TNF-α, and IL-1β. Meanwhile, the results confirmed that the excellent antioxidant activity of DWMPH, which was affected by the amino acid compositions, secondary structures, and MW distribution, was closely associated with the neuroprotective effect. The possible mechanism of DWMPH on alleviation of d-gal and AlCl_3_ induced neurotoxicity in mice is shown in Figure 6. The results above provide an idea that DWMPH might be a promising dietary supplement in protecting brain neurons against oxidative stress, because of their excellent antioxidant activity. The following works will explore the neuroprotective mechanism further and the absorption of DWMPH.

## Figures and Tables

**Table 1:** Initial body weight, final body weight, and food intake of each group. DWMPH—defatted walnut meal protein hydrolysates.

Group	Initial Body Weight (g)	Final Body Weight (g)	Food Intake (g/day)
Control	32.40 ± 1.88	43.10 ± 3.29	7.10 ± 1.24
d-gal+AlCl_3_	30.84 ± 2.36	41.93 ± 3.91	7.12 ± 0.41
d-gal+AlCl_3_+DWMPH	31.01 ± 2.74	43.70 ± 4.19	6.50 ± 0.43
DWMPH	32.65 ± 1.97	41.72 ± 3.97	6.23 ± 0.40

**Table 2:** Amino acid composition and essential amino acid evaluation of DWMPH.

Amino Acid	Content (g/100 g)		Amino Acid	Content (g/100 g)	
	DWMP	DWMPH		DWMP	DWMPH
Glu	24.239 ± 0.242a	23.495 ± 0.616a	Lys	2.911 ± 0.139a	3.536 ± 0.218a
Arg	15.048 ± 0.293a	13.874 ± 0.320b	Tyr	2.688 ± 0.203a	2.456 ± 0.252a
Asp	9.283 ± 0.569a	11.002 ± 0.430a	Thr	2.658 ± 0.123a	2.956 ± 0.209a
Leu	6.763 ± 0.166a	6.624 ± 0.144a	His	2.440 ± 0.200a	2.278 ± 0.114a
Val	5.050 ± 0.227a	4.869 ± 0.311a	Met	1.618 ± 0.196a	1.270 ± 0.163a
Gly	4.830 ± 0.223b	5.650 ± 0.134a	Trp	0.848 ± 0.064a	1.029 ± 0.130a
Phe	4.507 ± 0.178a	4.300 ± 0.248a	Cys	0.582 ± 0.074a	0.473 ± 0.066a
Ile	4.392 ± 0.352a	4.430 ± 0.235a	Met + Cys	2.199 ± 0.270a	1.742 ± 0.229a
Ala	4.152 ± 0.137a	4.316 ± 0.165a	Phe + Tyr	7.195 ± 0.025a	6.753 ± 0.004b
Ser	4.014 ± 0.173a	4.592 ± 0.163a			
Pro	3.980 ± 0.243a	2.857 ± 0.153b	EAA/TAA (%)	28.745 ± 0.267a	29.009 ± 0.423a

The values in a column with different letters indicate significant differences (*p* < 0.05).
